# Mast Cell-Derived Tryptase in Geographic Atrophy

**DOI:** 10.1167/iovs.17-22989

**Published:** 2017-11

**Authors:** D. Scott McLeod, Imran Bhutto, Malia M. Edwards, Manasee Gedam, Rajkumar Baldeosingh, Gerard A. Lutty

**Affiliations:** Wilmer Ophthalmological Institute, Johns Hopkins Hospital, Baltimore, Maryland, United States

**Keywords:** age-related macular degeneration, geographic atrophy, Bruch's membrane, RPE, choriocapillaris, choroid, degranulation, mast cells, tryptase, lipid

## Abstract

**Purpose:**

Our previous study demonstrated significantly more degranulating mast cells (MCs) in choroids from subjects with age-related macular degeneration compared to aged controls. This study examined the immunolocalization of tryptase, the most abundant MC secretory granule-derived serine protease, in aged control eyes and eyes with geographic atrophy (GA).

**Methods:**

Postmortem human eyes with and without GA were obtained from the National Disease Research Interchange. Tissue was fixed, cryopreserved, sectioned, and immunostained with a monoclonal antibody against tryptase. Sections were imaged on a Zeiss 710 Confocal Microscope.

**Results:**

In the posterior pole of all aged control eyes, tryptase was confined to choroidal MCs, which were located primarily in Sattler's layer. In eyes with GA, many MCs were located in the inner choroid near choriocapillaris and Bruch's membrane (BM). Tryptase was found not only in MCs but also diffusely around them in stroma, suggesting they had degranulated. In contrast with aged control eyes, eyes with GA also had strong tryptase staining in BM. Tryptase was observed within BM in regions of RPE atrophy, at the border of atrophy, and extending well into the nonatrophic region.

**Conclusions:**

Our results demonstrate that tryptase, released during choroidal MC degranulation, binds to BM in GA in advance of RPE atrophy. Tryptase activates MMPs that can degrade extracellular matrix (ECM) and basement membrane components found in BM. ECM modifications are likely to have a profound effect on the function and health of RPE and choroidal thinning in GA.

Mast cells (MCs) are key effector cells of inflammation and are present in connective tissue and at mucosal surfaces throughout the body. In their normal function, they contribute to tissue homeostasis, host defense, and tissue repair. Multiple receptors regulate the release of a vast array of proinflammatory mediators, proteases, and cytokines.^1,2^ However, when there is a repeated or long-term stimulus, MC activation leads to tissue damage and dysfunction. Consequently, MCs are implicated in the pathophysiologic aspects of numerous diseases, including asthma, anaphylaxis, malignancies, and cardiovascular diseases.^3,4^

Within seconds of stimulation, MCs can undergo degranulation, rapidly releasing preformed mediators present within cytoplasmic granules, including histamine, the proteases tryptase and chymase, and tumor necrosis factor-α. Tryptase is the most abundant serine proteinase in MCs. It has been estimated that up to 35 pg tryptase may be stored per cell compared to 2 pg histamine within secretory granules.^5^ It recently has been reported that tryptase is involved in the activation of pro-metalloproteases, like MMP-1, MMP-3, and MC tryptase is a gelatinase, similar to MMP-2 or MMP-9, and has potent gelatin degrading properties.^6,7^

In humans, MCs are abundant in the anterior and posterior uvea but absent in the retina.^8,9^ We reported previously that MC numbers and their degranulation are increased in age-related macular degeneration (AMD).^10^ This increase occurred in all forms of AMD, including early AMD. In this study, we further characterized choroidal MCs in human eyes and examined the distribution of secreted MC granule tryptase in aged control eyes and eyes with geographic atrophy.

## Materials and Methods

### Tissue Preparation

Human eyes were obtained from the National Disease Research Interchange (NDRI, Philadelphia, PA, USA). Use of this human tissue was in accordance with the Declaration of Helsinki with approval of the Joint Committee on Clinical Investigations at Johns Hopkins University School of Medicine. Eyes from five aged control subjects (mean age 84.4 ± 5.3 years) with no evidence of macular disease and from five subjects with geographic atrophy (GA; mean age 89 ± 5.9 years) were studied. All donors were Caucasian. The mean death to enucleation time was 4.72 ± 0.47 hours for controls and 4.08 ± 1.01 hours for GA, which was not significantly different according to Student's *t*-test (*P* = 0.24). The mean postmortem time for controls was 26.2 ± 2.77 hours, while that for GA subjects was 23.4 ± 2.7, which also were not significantly different (*P* = 0.145). GA was confirmed by reviewing limited ocular medical histories on the eye bank transmittal sheets and by postmortem gross examination of the posterior eyecup, using transmitted and reflected illumination on a dissecting microscope (Stemi 2000; Carl Zeiss Meditec, Inc., Thornwood, NY, USA), as we reported previously.^11^ GA was diagnosed if the there was a distinct area of RPE loss that had sharply defined borders upon gross examination of the eye cup. Tissues from one eye of each subject were cryopreserved ^12^ and frozen sections cut at 8 μm for immunofluorescence staining.

Whole choroids from the fellow eyes of three aged controls and three GA subjects were double labeled with antibodies against tryptase and chymase for whole mount analysis as previously described.^13^ Briefly, after taking gross photos of the eyecup before and after removing the retina, eyecups containing the choroid were soaked in 1% EDTA (disodium salt; dihydrate crystal; J.T. Baker, Inc., Phillipsburg, NJ, USA) in distilled water for 2 hours at room temperature to remove the RPE. Any adherent RPE cells were removed by squirting the choroid with EDTA solution from a syringe with a blunted 25-gauge needle. RPE-denuded choroids then were dissected from the sclera, washed briefly in 0.1 M cacodylate, and fixed overnight in 2% paraformaldehyde in 0.1 M cacodylate buffer at 4°C.

### Immunofluorescence in Sections

Immunofluorescence staining of sections was performed as previously described.^14^ Briefly, cryopreserved tissue sections from the posterior pole were permeabilized with absolute methanol at −20°C and blocked with 2% normal goat serum in Tris buffered saline (TBS; pH 7.4 with 1% BSA). After washing in TBS, the sections were incubated for 2 hours at room temperature with a mixture of primary antibodies (Table). After they were washed in TBS, sections were incubated for 30 minutes at room temperature with a cocktail of secondary antibodies containing 4′,6-diamino-2-phenyl-indole (DAPI). Sections then were treated with 1% Sudan black B in 70% ethanol for 10 minutes to quench RPE lipofuscin autofluorescence. Finally, sections were coverslipped with mounting medium (Vector Laboratories, Inc., Burlingame, CA, USA) and imaged with a Zeiss 710 confocal microscope (Carl Zeiss Meditec, Inc.). Some adjacent sections from those immunolabeled for tryptase were stained with oil red O as suggested by the supplier (Oil Red O Stain Kit; Polysciences, Warrington, PA, USA).

**Table:**
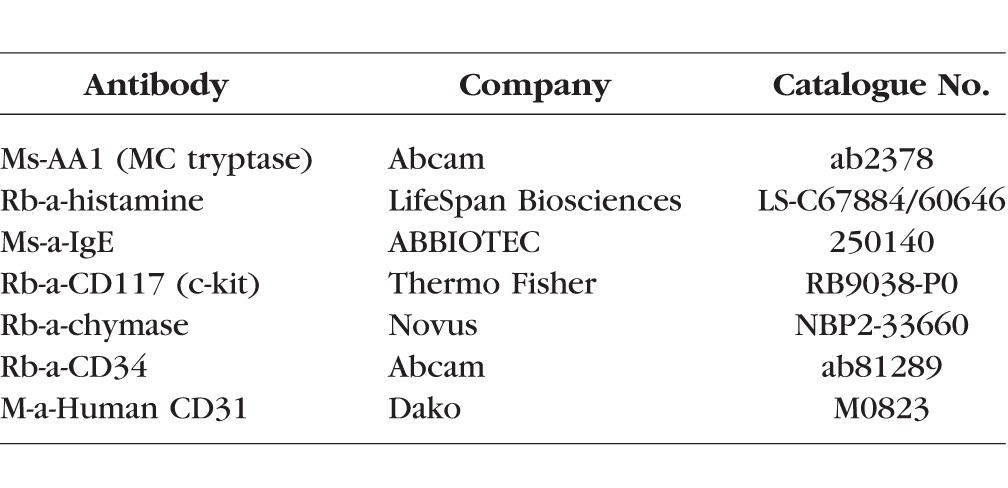
Antibodies Used for Immunohistochemistry

### Whole Mount Immunofluorescence

Choroids were washed twice in 0.1M cacodylate buffer at 4°C and then in TBS with 0.1% Triton X-100 (TBS-T) for 10 minutes. After washes, choroids were incubated with 5% normal goat serum in TBS-T with 1% BSA overnight at 4°C. Tissues then were washed with TBS-T and incubated with a mixture of mouse anti MC tryptase antibody (ab2378,1:500; Abcam, Inc., Cambridge, MA, USA) and rabbit anti-MC chymase antibody (NBP2-33660, 1:500; Novus Biologicals, Littleton, CO, USA) for 72 hours at 4°C. Following three washes in TBS-T, tissues were incubated for 48 hours at 4°C with secondary antibodies (1:200): goat anti-mouse conjugated with Cy3 (Jackson ImmunoResearch, West Grove, PA, USA); goat-anti rabbit conjugated Alexafluor647 (A647; Invitrogen, Carlsbad, CA, USA); and UEA lectin/FITC conjugated (1:100; Cat#GTX01512; GeneTex, Inc., Irvine, CA, USA). Tissues were washed in TBS and then imaged with a Zeiss LSM 710 confocal microscope (Carl Zeiss Meditec, Inc.) at 488, 561, and 633 nm excitation (FITC, Cy3, A647 staining, respectively).

### Image Analysis for Cell Counts

Maximum intensity projection, ×5 stitched images were exported from Zen Software as full resolution Tiff images and opened in Adobe Photoshop (CS6, Adobe Systems Incorporated, San Jose, CA, USA). Three 1204 × 1204 pixel dimension selections (equivalent to 1 mm^2^) were made randomly of regions in the submacular choroid and pasted into new image documents. Each image was adjusted using the channel mixer function in Photoshop to segregate the different labels (blue for europaeus agglutinin [UEA]-labeled blood vessels, red for tryptase, and green for chymase). Each channel was copied and saved as separate Tiff images. Levels and thresholding then were adjusted and saved for analysis in ImageJ (National Institutes of Health [NIH], Bethesda, MD, USA; available in the public domain at https://imagej.nih.gov/ij/). The images were converted to binary, noise reduction applied, and the analyze particle function used to perform cell counts. The cutoff used for the pixel dimension of the particles counted was 50-infinity.

## Results

### Tryptase/Chymase Double-Labeling of Whole Mounts

**Figure 1:**
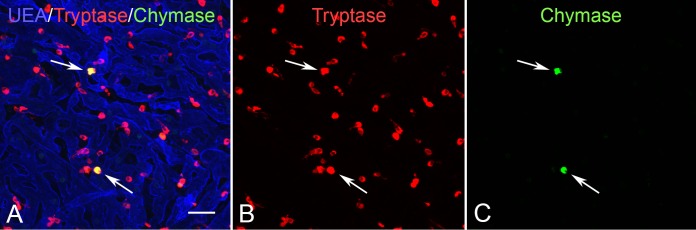
Aged control whole mount choroid immunolabeled for (A) UEA lectin (blue), (B) tryptase (red), and (C) chymase (green). The vast majority of human choroidal MCs are of the MC_T_ phenotype (tryptase^+^/chymase^−^). Only a small percentage of MCs are MC_tc_ (tryptase^+^/chymase^+^) (arrows). Scale bar: 30 μm.

**Figure 2:**
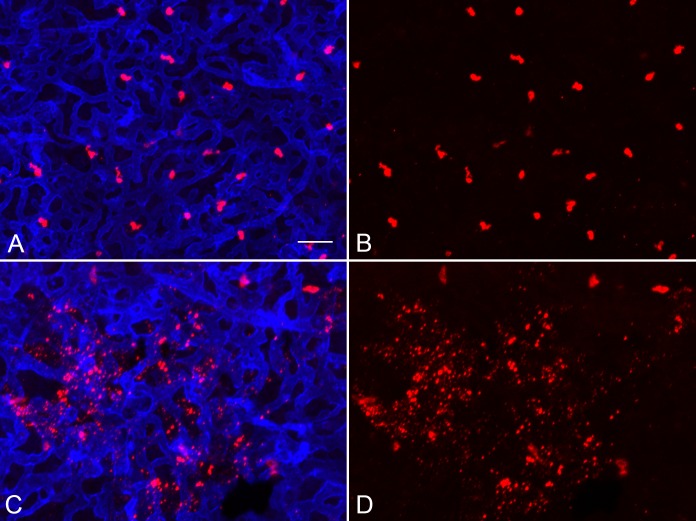
Aged control (A, B) and GA (C, D) whole mount choroids immunolabeled with UEA lectin (blue) and anti-tryptase (red). MC degranulation near the border region of RPE atrophy demonstrates the association between MC degranulation and choriocapillaris atrophy. The free tryptase^+^ granules were at the level of the inner aspect of the choriocapillaris as determined in z stacks. Scale bar: 30 μm.

Cell counts in double-labeled whole mounts revealed the vast majority of MCs (98%) in human choroid express only tryptase (T MCs or MC_T_ type) with very few expressing tryptase and chymase (TC MCs or MC_TC_ type; Fig. 1). This was true for aged controls and GA eyes. In control eyes, very few MCs appeared degranulated, based on tryptase^+^ immunolabeling being restricted to the cell cytoplasm (Figs. 2A, 2B). However, in GA choroids, free tryptase^+^ granules were evident in nonatrophic regions, near the border of atrophy (Figs. 2C, 2D) and in regions with RPE atrophy. Examination of Z Stacks demonstrated that most of these free tryptase^+^ granules in GA choroid were present near the inner surface of the choriocapillaris, not at the level of MCs in Sattler's and Haller's layer. They varied considerably in size and were spherical in shape.

### Tryptase Localization in Sections of Control and GA Eyes

**Figure 3:**
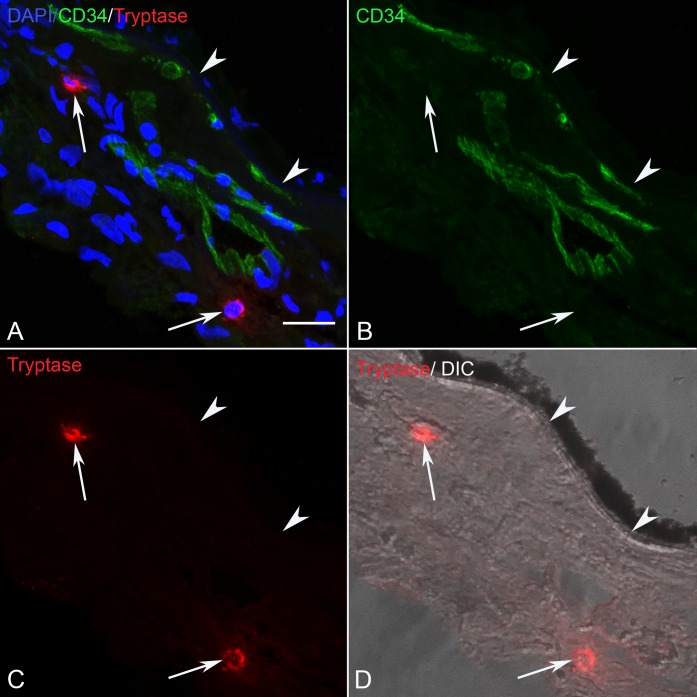
(A–D) Section from an 85-year-old aged control subject showing (C) tryptase^+^ MCs (arrows), (B) CD34+ blood vessels (green) and BM (arrowhead). Most MCs in aged control eyes were located in Sattler's and Haller's layer where tryptase^+^ granules were confined to MC cytoplasm. In (A) the colors are merged and (D) is differential interference contrast (DIC) illumination so RPE can be distinguished. Scale bar: 20 μm.

**Figure 4:**
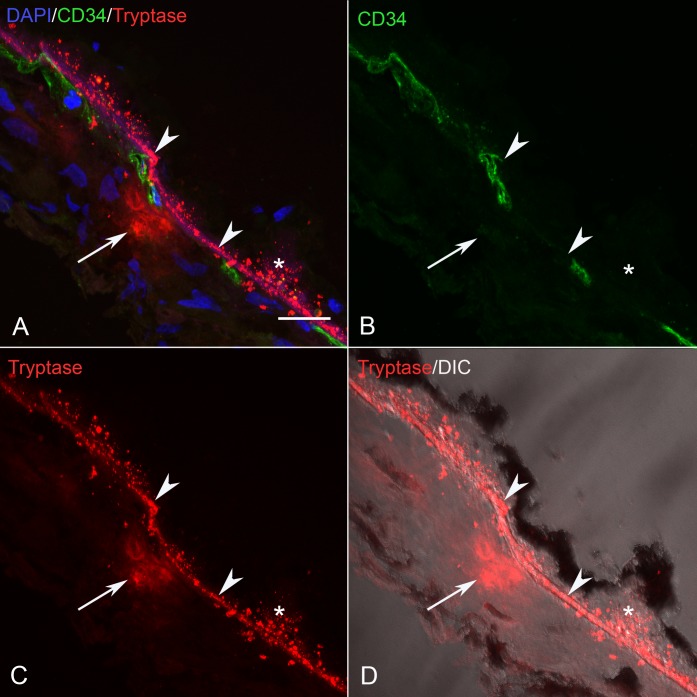
(A–D) Section from an 89-year-old GA subject showing (C) a degranulated tryptase^+^ MC (arrow), (B) CD34^+^ blood vessels and BM (arrowhead) in a nonatrophic region of RPE (*). (D) DIC illumination and (A) has all colors merged. MCs in GA eyes often were located near the choriocapillaris where diffuse tryptase immunoreactivity was observed in the surrounding choroidal stroma. Granular tryptase reaction product was localized in BM (arrowhead), sub RPE deposits and drusen. Scale bar: 20 μm.

**Figure 5:**
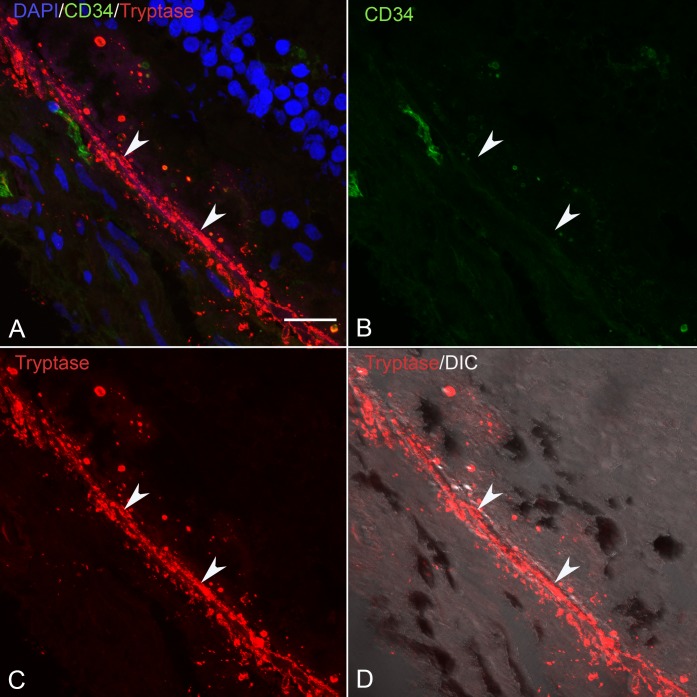
(A–D) Section from the same 89-year-old GA subject in shown in Figure 4 near the border of RPE atrophy demonstrates granular tryptase immunoreactivity in BM (arrowhead). (B) The density of CD34^+^ choriocapillaris is reduced in this border region and (C) RPE are multilayered with granular tryptase immunoreactivity around them and within BM. (D) DIC illumination and (A) has all colors merged. Scale bar: 20 μm.

**Figure 6:**
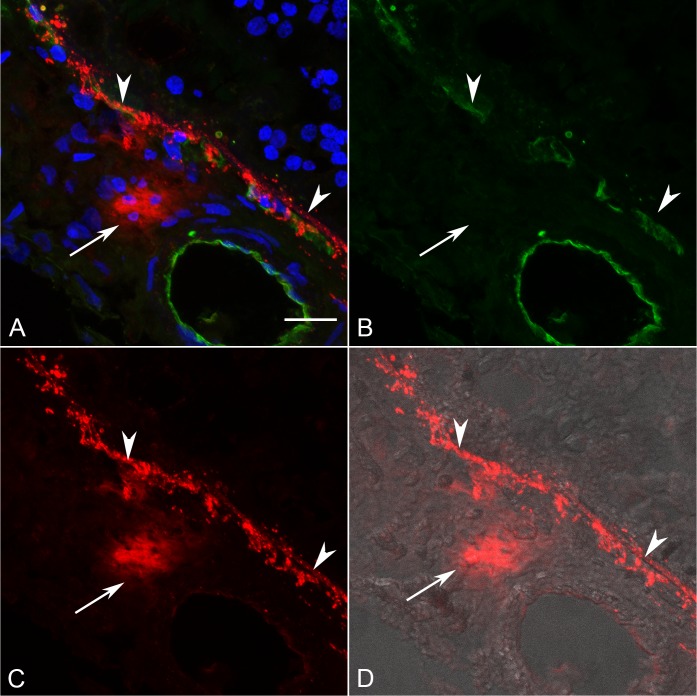
(A–D) Section from the same 89-year-old GA subject shown in Figure 5 in a region with RPE atrophy showing, (B) reduced CD34+ choriocapillaris density and diffuse tryptase staining (C) surrounding a degranulating MC (arrow) and granular tryptase immunoreactivity in BM (arrowhead). (D) DIC and (A) has all colors merged. Scale bar: 20 μm.

In sections of aged control eyes, tryptase immunoreactivity generally was confined to granules in MC cytoplasm residing in Sattler's and Haller's layer (Fig. 3; Supplementary Fig. S1). Few MCs appeared degranulated. In almost all control subjects, Bruch's membrane (BM) and the RPE were unlabeled. However, in one control choroid, there were a few regions in the vicinity of choriocapillaris and BM with a few tryptase^+^ granules (Supplementary Fig. S2). In all GA eyes, tryptase immunoreactivity was observed in and diffusely surrounding MCs of choroidal stroma in regions with nonatrophic RPE (Fig. 4; Supplementary Fig. S3). The diffuse tryptase staining around the MCs suggests they were degranulated. Immunolabeling also was present in BM, drusen, and RPE in GA eyes where the staining pattern appeared as rounded particulate bodies. This also was the case at the border region (Fig. 5; Supplementary Fig. S4) and in the atrophic regions (Fig. 6; Supplementary Fig. S5) of all GA eyes. These particulate bodies varied considerably in size and averaged 2.2 μm^2^ in area. Additionally, tryptase immunolabeling was observed in the walls of some arteries in GA choroid (Supplementary Figs. S5, S6).

### Histamine, cKit and IgE Localization in Control and GA Eyes

**Figure 7:**
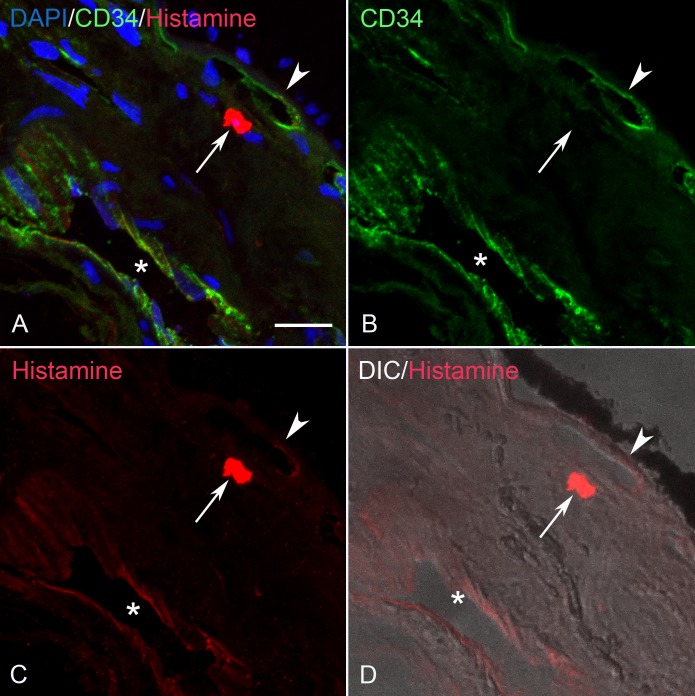
(A–D) Section from an 86-year-old aged control subject showing (C) a histamine^+^ MC (arrow), (B) CD34^+^ blood vessels and BM (arrowhead). (D) DIC and (A) has all colors merged. The endothelium of an artery is weakly histamine^+^ (*). BM (arrowhead) is unlabeled D. Scale bar: 20 μm.

**Figure 8:**
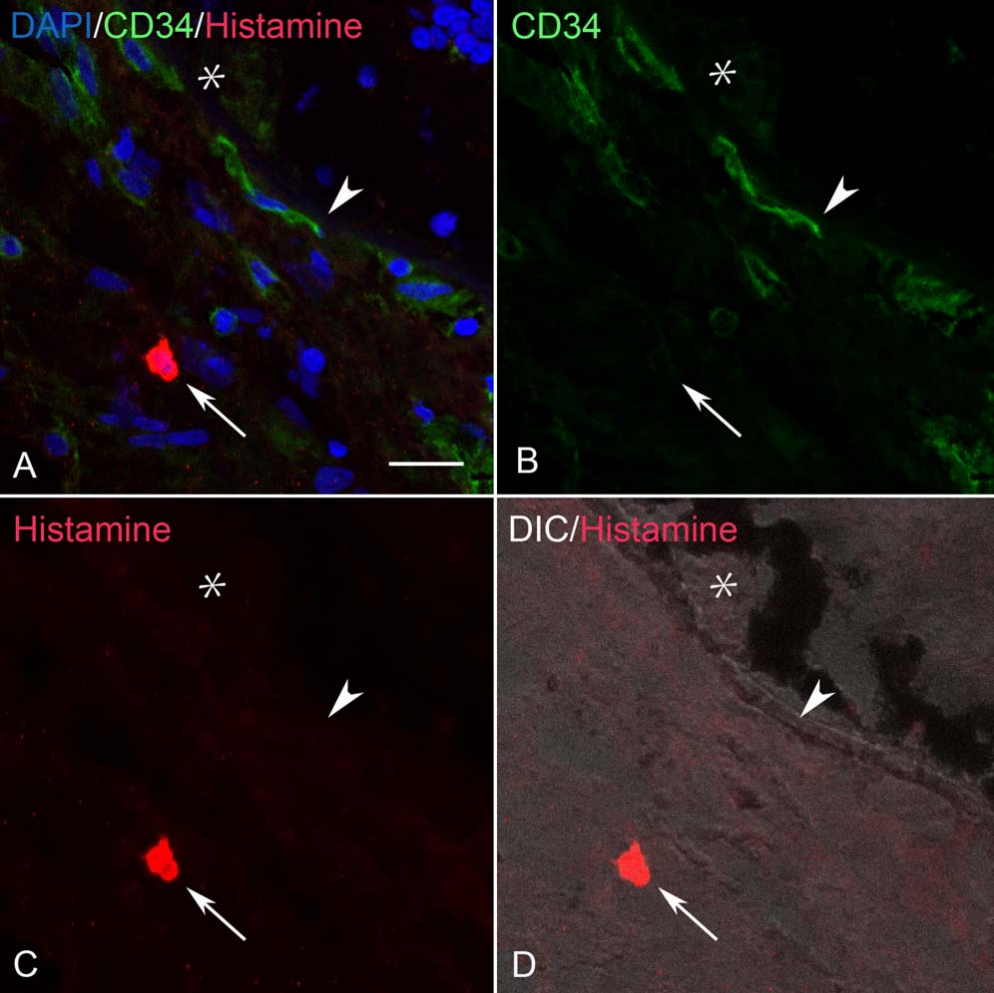
(A–D) Section from an 89-year-old GA subject near the border of RPE atrophy showing (C) a histamine^+^ MC (arrow), (B) CD34^+^ blood vessels, and BM (arrowhead). (D) DIC illumination shows a drusen^+^ (*), and (A) has all colors merged. BM (arrowhead), drusen (*) and RPE that are unlabeled. Scale bar: 20 μm.

**Figure 9:**
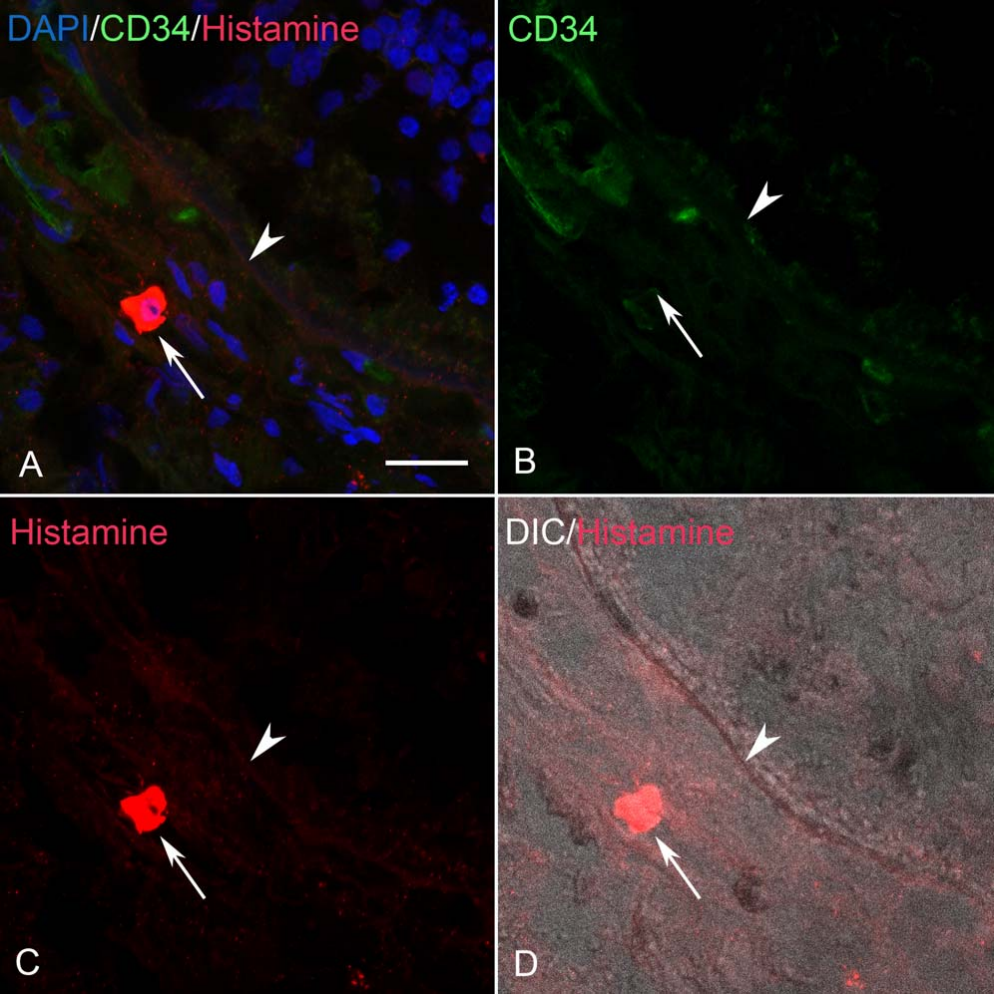
(A–D) Section from the same 89-year-old GA subject shown in Figure 8 in a region of RPE atrophy showing (C) a histamine^+^ MC (arrow), (B) CD34^+^ blood vessel attenuation, and unstained BM (arrowhead). (D) DIC illumination and (A) has all colors merged. Scale bar: 20 μm.

In aged control eyes, histamine, like tryptase, was localized primarily to choroidal MC cytoplasm (Fig. 7). In some cases, weak immunolabeling also was observed in the endothelium of some choroidal blood vessels. In GA eyes, histamine also was confined primarily to the cytoplasm of MCs in all regions (nonatrophic, border of atrophy, and atrophic; Figs. 8, 9). Unlike tryptase, no immunostaining was observed in BM, drusen, or RPE. There were no differences in the staining patterns between aged control and GA eyes with antibodies to IgE and cKit where both were confined strictly to MCs (data not shown). As was the case with histamine, no immunostaining of BM, drusen, or RPE was observed.

### Oil Red O Staining

**Figure 10:**
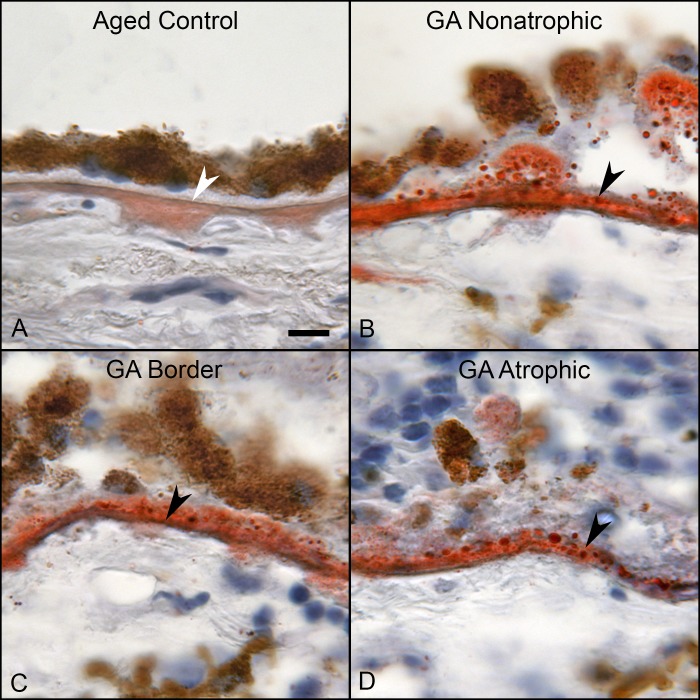
Sections from an 81-year-old aged control subject (A) and an 89-year-old GA subject (B–D) showing oil red O staining. In aged controls, weak diffuse staining was observed in BM. In the GA eyes, intense staining was observed in BM and drusen. The staining was granular in appearance and was remarkably similar to tryptase immunoreaction product in GA. Scale bar: 10 μm.

**Figure 11:**
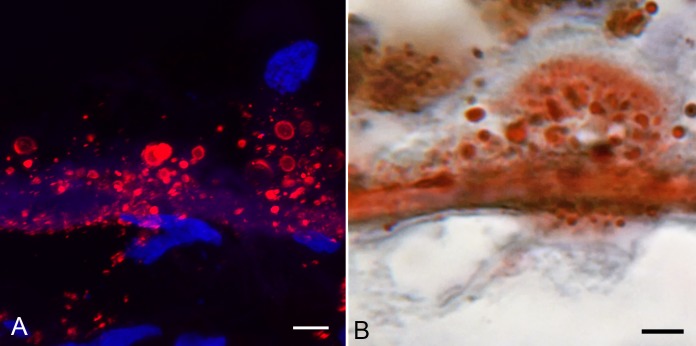
Semiserial sections of a 90-year-old GA choroid comparing tryptase localization (A) with oil red O staining (B) in BM and drusen. Note the rounded particulate bodies are labeled in both and reside in the same tissue compartment. Scale bar: 5 μm.

In aged control eyes, oil red O lightly stained BM and some intercapillary septa (Fig. 10A). The staining was even and diffuse. In GA eyes, oil red O staining was intense in BM and drusen (Figs. 10B–D). The staining had a granular appearance resembling the particulate bodies immunolabeled with tryptase. The localization, size ( 3 ± 0.8 μm^2^), and spherical shape of these lipid bodies in oil red O–stained sections were remarkably similar to those seen in tryptase-stained sections (Fig. 11). The lipid bodies also were present in the walls of arteries, which also were immunolabeled with tryptase (data not shown).

## Discussion

MCs are derived from multilineage hematopoietic progenitors that migrate to vascularized tissues and organs, where they mature and ultimately reside. Activation, via a variety of mechanisms, triggers degranulation of MCs, releasing histamine, cytokines, chemokines, and proteases into the milieu. There are two phenotypes of human MCs: T MCs contain only tryptase, and are found predominantly in alveolar walls and the small intestinal mucosa. TC MCs containing tryptase and chymase are present predominantly in the dermis of the skin, intestinal submucosa, and around blood vessels.^5^ It has been reported previously that MCs of human choroid belong to the TC subtype with granules containing chymase and tryptase.^15^ Our current study, using double labeling with antibodies against tryptase and chymase in choroidal whole mounts, found that only approximately 2% of human choroidal MCs express chymase. The reason for the discrepancy in our study and the previous study is clear. The study by May used a combined enzyme histochemical-immunohistochemical technique with an antibody against tryptase together with nonspecific esterase enzyme histochemical activity using naphthol-AS-D chloroacetate as the substrate to distinguish between the phenotypes. The nonspecific esterase activity using the technique of Leder^16^ is not specific to MCs nor does it label chymase specifically.

In a previous study,^10^ we found that there were increased numbers of MCs in choroids from human eyes with AMD compared to aged controls. Additionally, we found a significant increase in the number of degranulating MCs in AMD eyes. The current study extends our previous observations by examining the localization of tryptase, the most prominent protease in human choroidal MCs, in cryosections and flat mounts of aged control and GA choroids. The diffuse tryptase immunostaining seen in choroidal stroma surrounding numerous MCs in GA, which was much less common in control eyes, confirms our previous findings of increased MC degranulation in these eyes. Perhaps the most striking finding in all GA eyes was the localization of tryptase, in the form of rounded particles, in BM. No association was observed with histamine immunostaining and these structures.

Oil red O staining suggests that the rounded particles of tryptase immunoreactivity in GA choroid might be low density lipoprotein (LDL) deposited in BM, RPE, and drusen. The accumulation of lipids as a consequence of aging and AMD has been well characterized by Curcio et al.^17–20^ There is considerable indirect evidence suggesting a connection between lipid deposition/oxidation and aging diseases, such as AMD. Accumulation of oxidized lipids in RPE, BM, and CH in the aging eye impedes the proper flow of nutrients and metabolites. It also is a likely cause of chronic inflammation. It has been demonstrated that LDL binds to released MC granules and forms complexes.^21^ Heparan, which is stored in complex with tryptase in the secretory granule and cosecreted during degranulation, is essential for binding of LDL to granules.^22,23^ Tryptase is a unique serine protease that is active enzymatically only in its tetrameric form.^24^ It requires glycosaminoglycans (GAGs), notably heparan proteoglycan of MCs, for preservation of its enzymatic activity.^25^ The heparan proteoglycans tend to prevent monomerization of the enzyme after secretion, preserving its activity for a prolonged period of time in the extracellular space. In addition to the fibrous connective tissue elements present in BM, a variety of proteoglycans also are found in this compartment. The major proteoglycans present are the chondroitin sulfate, dermatan sulfate, and heparan sulfate-types.^26^ In addition, the nonsulfated glycosaminoglycan, hyaluronic acid also is present in this compartment. With age, there is an increase in proteoglycans in BM.^27^ Proteoglycans have a well-established role of retaining LDLs early in the pathogenesis of atherosclerosis.^28^ Like MC–derived heparan proteoglycans, the arterial proteoglycans also can stabilize the tryptase tetramer or may modulate the activity of tryptase. Degranulated MCs present in atherosclerotic lesions release tryptase and the released tryptase is stabilized by arterial proteoglycans.^3^ Taken together, the accumulation of LDL and increased proteoglycans within BM with age^29,30^ may act in concert to bind and stabilize secreted MC tryptase and to preserve the enzymatic activity in this structure for prolonged periods.

Studies in aging skin suggest that MC-derived proteases can activate MMPs. It has been reported that tryptase is involved in the activation of pro-metalloproteases, like MMP-1, MMP-3, and pro-uPA, into their active forms.^31^ Moreover, recent data indicated that MC tryptase is a gelatinase, similar to MMP-2 or MMP-9, and has potent gelatin degrading properties. Tryptase, either alone or in conjunction with activation of MMPs, can participate in extracellular matrix (ECM) damage, including collagen I and IV degradation (Iddamalgoda et al.^31^) and, therefore, possibly the destruction of BM and choroidal stroma.

The deleterious effect of tryptase on epithelial basement membranes has been documented in many organ systems. Tryptase induces intestinal permeability by degrading the epithelial basement membrane.^32,33^ Zhong et al.^34^ have shown that the modification of the epithelial basement membrane reduces ZO-1 and occludin making the epithelial barrier permeable. Iddamalgoda et al.^31^ have shown that tryptase will degrade collagen IV and activate MMP9 in dermis, resulting in basement membrane degradation in dermis. Therefore, it seems logical that tryptase observed in BM may be degrading it, causing disruption of RPE cell outer barrier properties and perhaps loss of these cells from BM. This hypothesis was investigated in vitro by Arai et al.^35^ using ARPE19 cells. They report that tryptase did not cause loss of junctional complexes but did enhance migration of RPE in a wound assay. The source of tryptase and its activity in this experiment is not clear nor is it clear that these RPE monolayers had made a basement membrane, which tryptase could degrade. Furthermore, this is a very acute exposure of tryptase (24–48 hours), whereas in GA it appears that tryptase may be present chronically and may be stabilized and preserved by its binding to heparan and lipids in BM.

In conclusion, choroidal MC tryptase is localized prominently to BM in geographic atrophy subjects. One scenario for the role of MCs and their tryptase in GA could be that MC degranulation and release of tryptase granules is stimulated by elevated C3a, C5a, C-reactive protein (CRP), and AGEs found in GA choroid.^36–38^ AGEs promote lipoprotein retention in BM^30^ and tryptase binds to lipoproteins, which stabilize the enzyme.^22^ This protease, for which there appears to be no natural inhibitors, could be present chronically in BM, in choroidal vascular basement membranes in atherosclerotic blood vessels, and in choroidal stroma which has AGEs. Degradation of basement membranes could result in loss of RPE barrier properties as it does in intestine, RPE migrating from BM, loss of choroidal endothelial cells, and thinning of choroid by degradation of stroma. MC degranulation is increased significantly in early and intermediate AMD^10^ and tryptase was observed in BM in nonatrophic regions in GA subjects in our study. Based on our results, inhibition of tryptase could be a potential therapeutic target during early AMD and also may be effective in slowing progression of GA atrophic regions.^39^

## Supplementary Material

Supplement 1
