# Supplementary material for: Mast Cell-Derived Tryptase in Geographic Atrophy
Source: Invest Ophthalmol Vis Sci. 2017 Nov;58(13):5887–96. doi: 10.1167/iovs.17-22989 (PMC5699534; doi:10.1167/iovs.17-22989)
Supplement: Supplement 1 [file iovs-58-13-03_s01-s06.pdf]

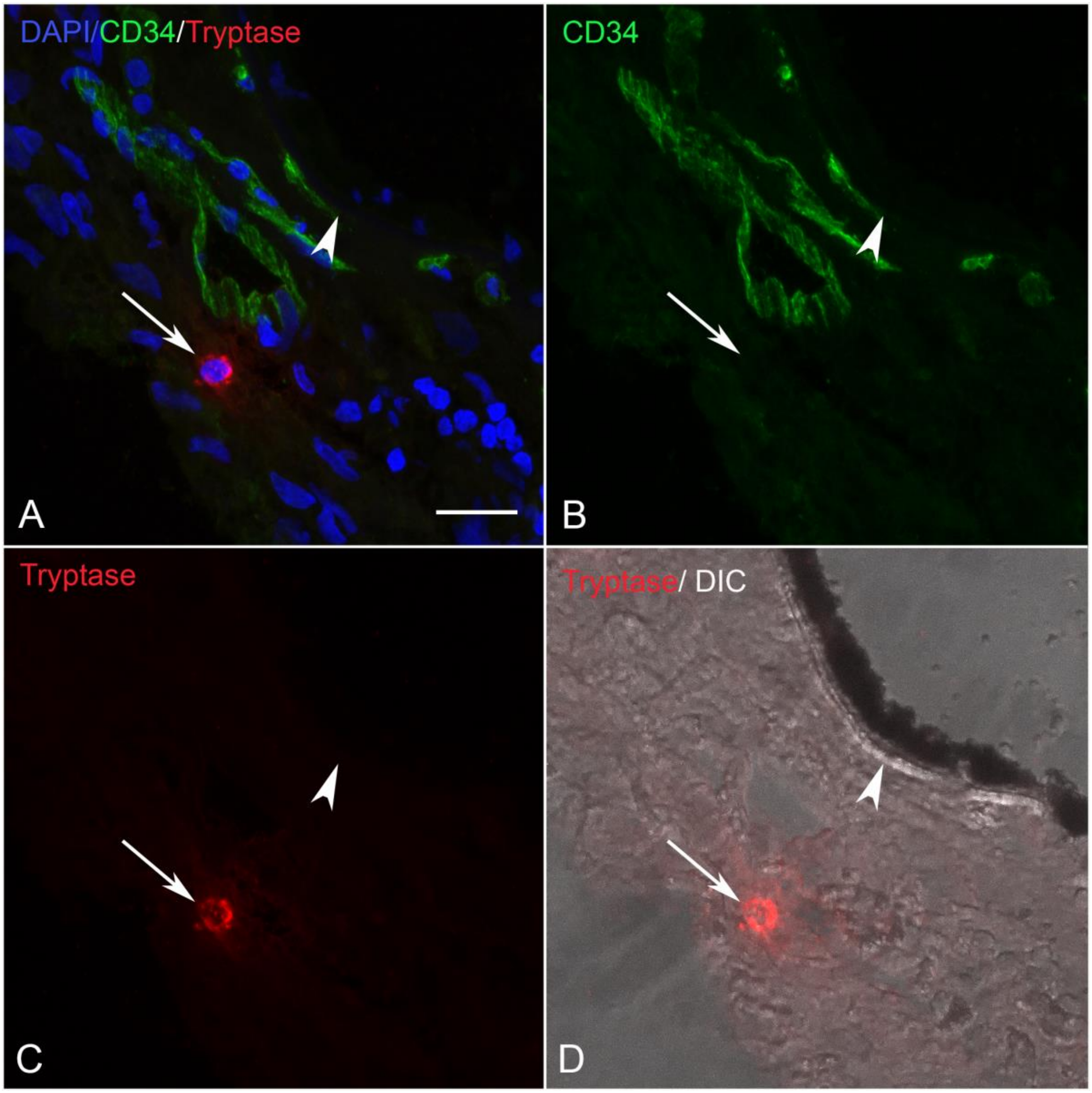

Supplemental Figure 1: Section from an 81 year old aged control subject showing a tryptase^{+} mast cell (arrow), CD34+ blood vessels (green) and Bruch's membrane (arrowhead). As shown in Figure 3, the majority of mast cells in aged control eyes were located in Sattler's and Haller's layer where tryptase^{+} granules were confined to mast cell cytoplasm. (D) is DIC illumination of the same area.Scale bar = 20μm

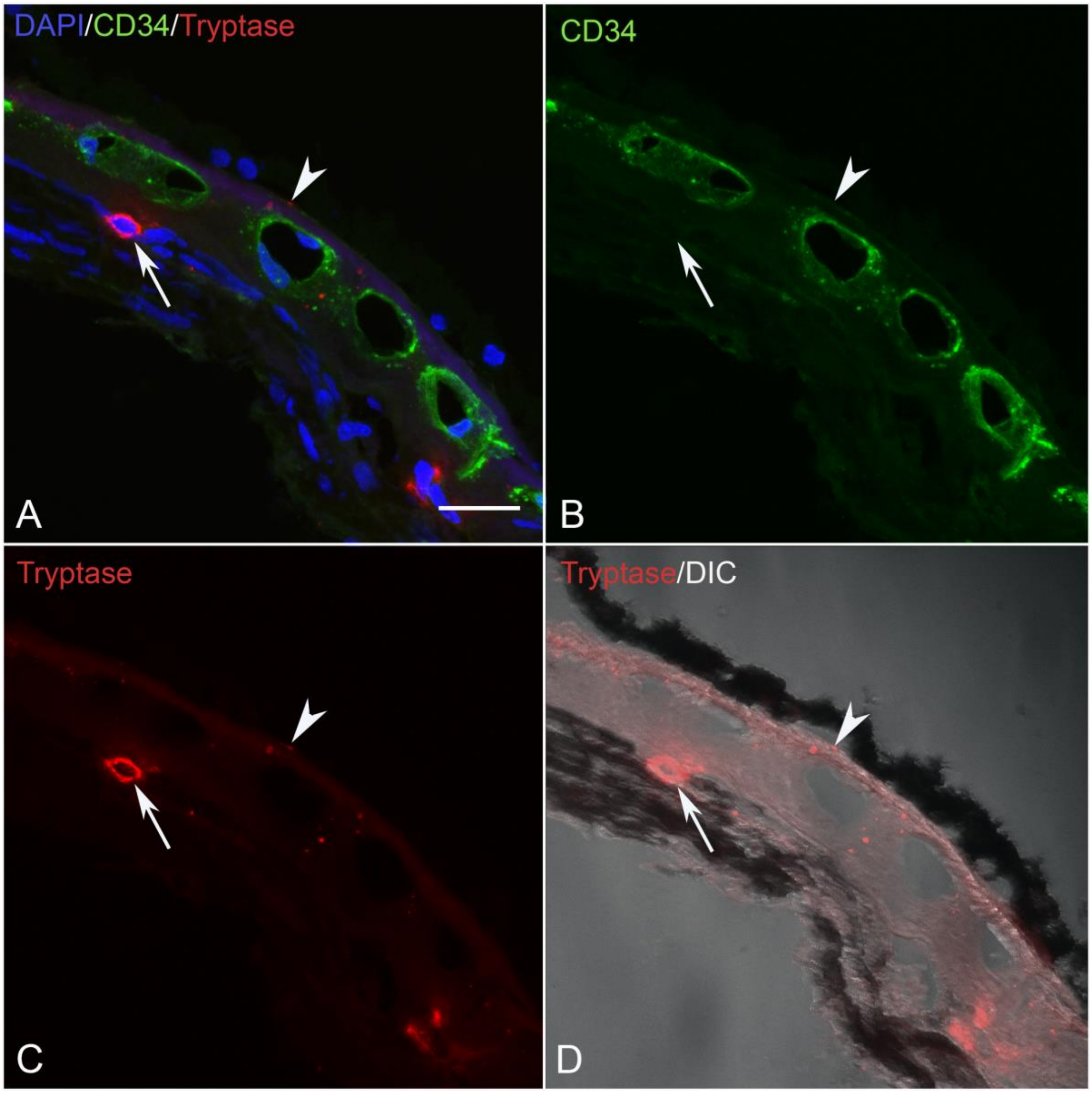

Supplemental Figure 2: Section from an 86 year old aged control subject showing a tryptase$^+$ mast cell (arrow), CD34$^+$ blood vessels and Bruch's membrane (arrowhead) where a few tryptase$^+$ granules were observed. Scale bar = 20μm

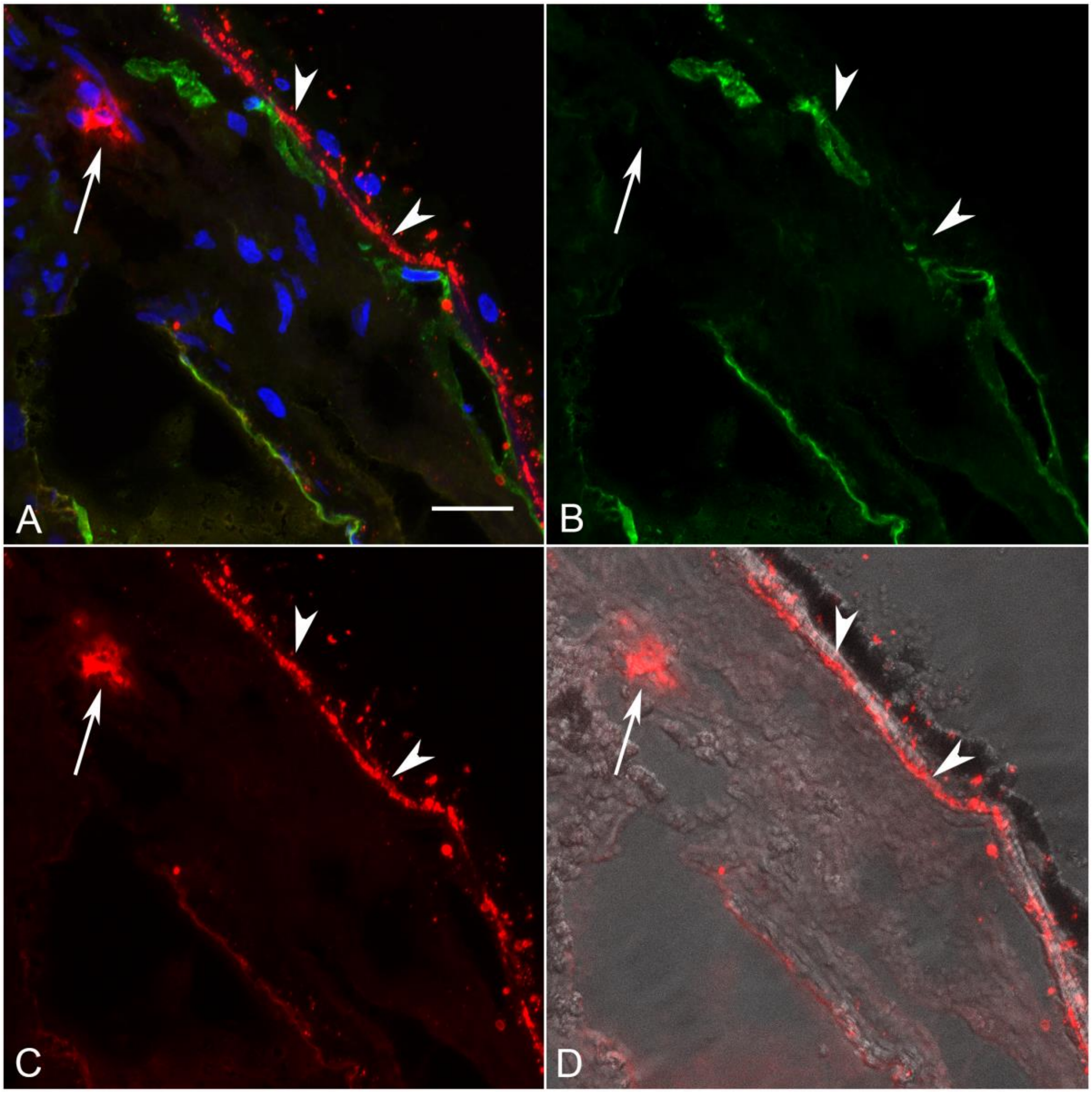

Supplemental Figure 3: Section from an 90 year old GA subject showing a degranulating tryptase^{+} mast cell (arrow), CD34^{+} blood vessels and Bruch's membrane (arrowhead) in a nonatrophic region of RPE. Diffuse tryptase immunoreactivity was observed in the choroidal stroma surrounding mast cells and granular tryptase reaction product was localized in Bruch's membrane (arrowheads) and in RPE. Scale bar = 20μm

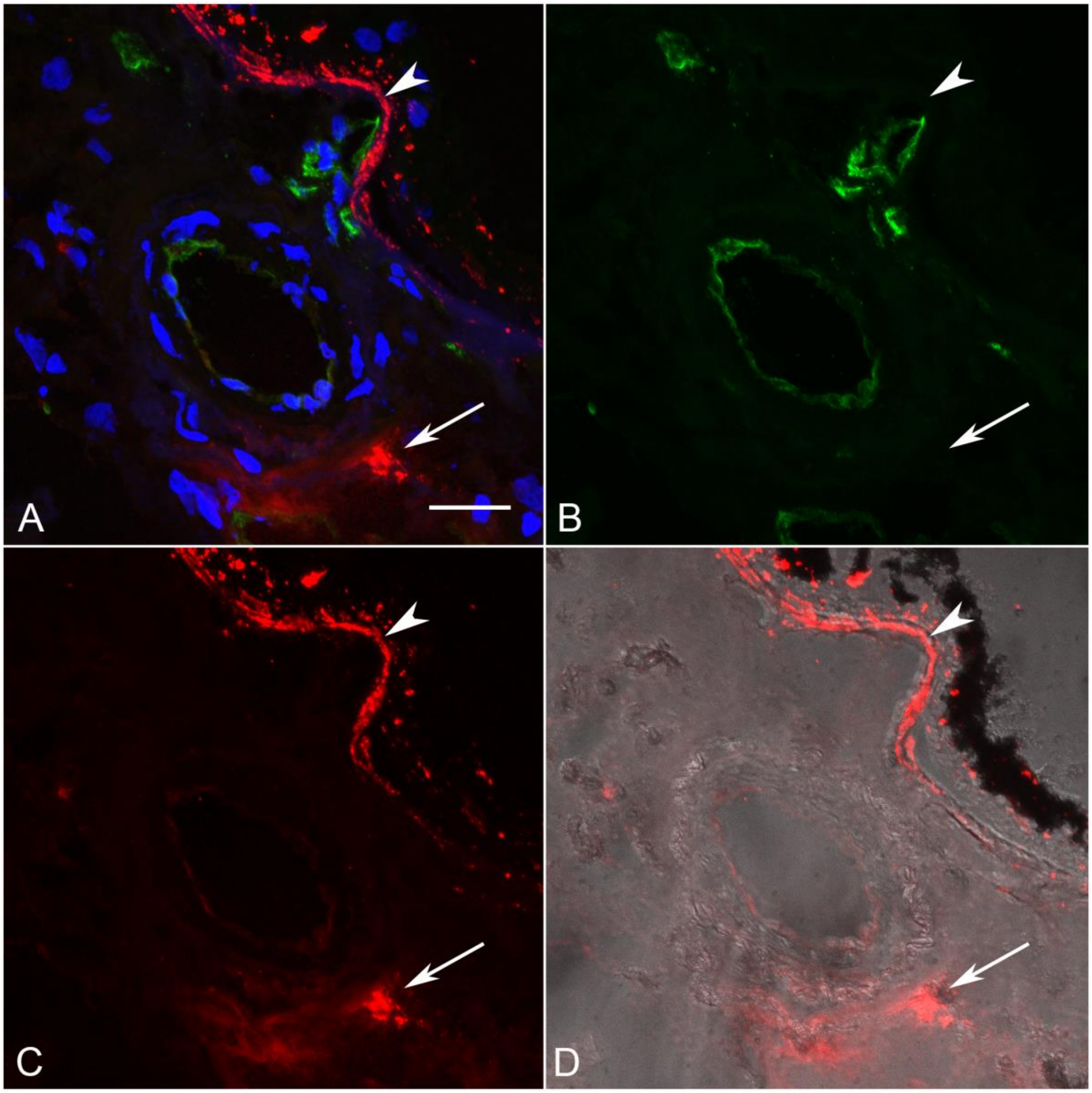

Supplemental Figure 4: Section from the same 90 year old GA subject shown in supplemental Figure 3 near the border of RPE atrophy showing diffuse tryptase surrounding a degranulating mast cell and granular tryptase immunoreactivity in Bruch's membrane (arrowhead). The density of $CD34^+$ choriocapillaris is reduced in this border region and RPE appear to contain granular tryptase reaction product. Scale bar = 20μm

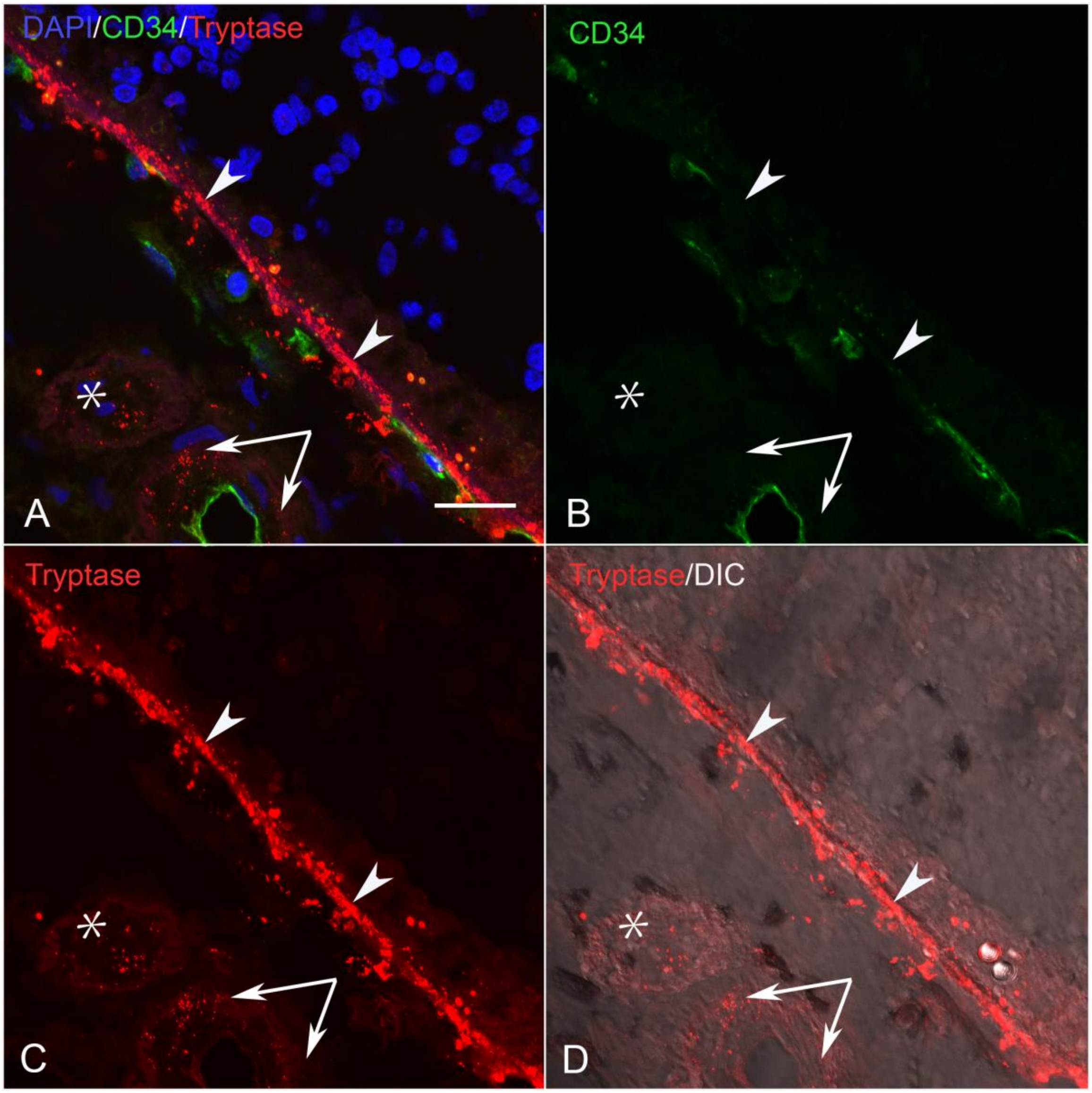

Supplemental Figure 5: Section from the same 90 year old GA subject shown in supplemental Figure 4 in a region with RPE atrophy showing granular tryptase immunoreactivity in Bruch's membrane (arrowhead), around the surviving viable choriocapillaris (green), in the thickened wall of an artery (paired arrows) and in a non-endothelialized ($CD34^-$) arterial wall (*). Scale bar = 20μm

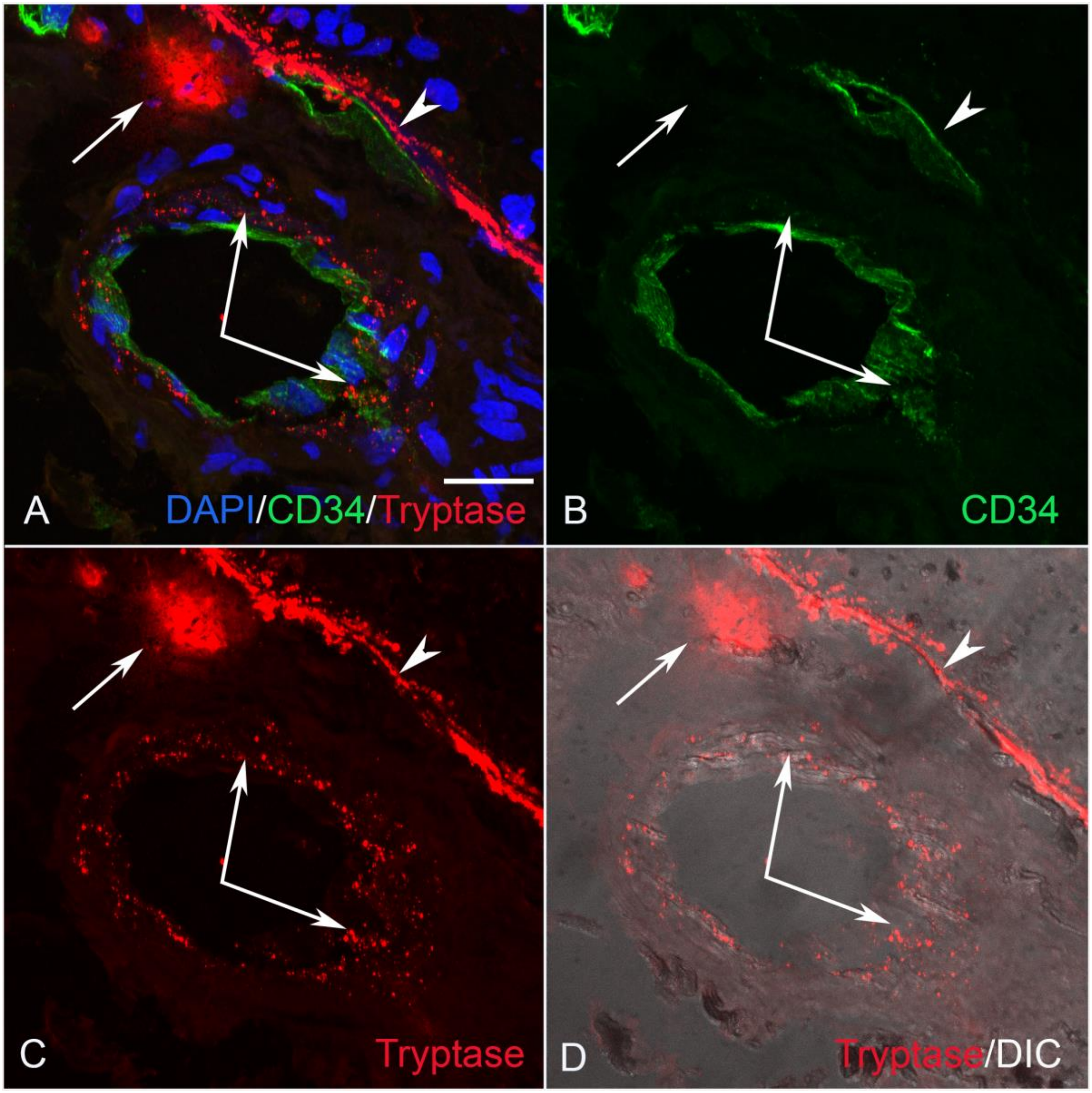

Supplemental Figure 6: Section from the same 90 year old GA subject shown in supplemental Figure 4&5 in a region with RPE atrophy showing diffuse tryptase staining surrounding a degranulating mast cell (arrow) and granular tryptase immunoreactivity in Bruch's membrane (arrowhead) and the thickened wall of a choroidal artery (paired arrows). The density of $CD34^{+}$ choriocapillaris is reduced in regions with RPE atrophy. Scale bar = 20μm
